# Exploration of Neuroanatomical Characteristics Relating to Progression from Preclinical to Prodromal Alzheimer's Disease

**DOI:** 10.1002/alz.087202

**Published:** 2025-01-09

**Authors:** Hak Hyeon Kim, Min Jeong Kwon, Sungman Jo, Jieun Park, Jiwon Kim, Jae Hyoung Kim, Sang Eun Kim, Ki Woong Kim, Ji Won Han

**Affiliations:** ^1^ Seoul National University Bundang Hospital, Seongnam Korea, Republic of (South); ^2^ Seoul National University College of Medicine, Seoul Korea, Republic of (South); ^3^ Seoul National University College of Natural Sciences, Seoul Korea, Republic of (South); ^4^ Seoul National University, Seoul Korea, Republic of (South); ^5^ Center for Nanomolecular Imaging and Innovative Drug Development, Advanced Institutes of Convergence Technology, Suwon Korea, Republic of (South)

## Abstract

**Background:**

To investigate the neuroanatomical characteristics at the whole‐brain level associated with progression from amyloid‐positive preclinical to prodromal Alzheimer’s disease (AD) in relation to amyloid deposition and regional atrophy.

**Method:**

We included 45 participants with amyloid‐positive preclinical AD and 135 participants with prodromal AD matched 1:3 by age, sex, and education, from participants in the Korean Longitudinal Study on Cognitive Aging and Dementia and visitors to the dementia clinic of Seoul National University Bundang Hospital. All participants underwent ^18^F‐florbetaben positron emission tomography and 3D structural T1‐weighted magnetic resonance imaging. We calculated the standardized uptake value ratios (SUVRs) and volumes in 80 regions of interest (ROIs) using the Desikan‐Killiany‐Tourville atlas and compared them between the preclinical and prodromal AD groups. We used the least absolute selection and shrinkage operator (LASSO) logistic regression model to identify ROIs associated with prodromal AD in relation to amyloid deposition, regional atrophy, and their interaction.

**Result:**

Although global SUVR did not differ between the amyloid‐positive preclinical and prodromal AD groups, the prodromal AD group showed a higher SUVR in the superior parietal cortex, right precuneus cortex, and right supramarginal gyrus. Regional volume differences between the two groups were observed in the overall cortex and subcortical areas, except in the occipital lobe, and the LASSO logistic regression model showed significant associations between prodromal AD and atrophy in the entorhinal cortex, inferior parietal cortex, both amygdalae, and left hippocampus. The mean SUVR in the right superior parietal cortex (beta coefficient = 0.0172) and its interaction with the regional volume (0.0672) were also selected in the LASSO model. The mean SUVR in the right superior parietal cortex was strongly associated with an increased risk of prodromal AD, particularly in participants with lower regional volume.

**Conclusion:**

Compared with amyloid‐positive preclinical AD, patients with prodromal AD showed more regions with significant volume differences than amyloid deposition. Amyloid deposition in the right superior parietal cortex may play a significant role in the progression of prodromal AD, particularly in interaction with its reduced volume.

